# Detection of polio virus in Gaza's sewage demands immediate action

**DOI:** 10.3389/fpubh.2024.1474554

**Published:** 2024-12-03

**Authors:** Tara P. Menon, Kate Zinszer, Samer Abuzerr

**Affiliations:** ^1^Virginia Tech Carilion School of Medicine, Roanoke, VA, United States; ^2^Department of Social and Preventive Medicine, School of Public Health, University of Montreal, Montréal, QC, Canada; ^3^Department of Medical Sciences, University College of Science and Technology, Khan Younis, Palestine

**Keywords:** polio, sewage, public health, detection, Gaza

## Introduction

The recent discovery of type 2 vaccine-derived poliovirus (VDPV2) in sewage water samples from Khan Younis and Deir al Balah in Gaza is a critical public health emergency demanding immediate intervention (1). This finding exposes thousands of Gazans, particularly those living in densely populated displaced persons' camps, to this highly infectious disease, which can cause severe deformities and paralysis. There is no cure for polio; vaccination is the most effective way to prevent infection and subsequent transmission. The aim of this report is to underscore the urgency of intervention and draw attention from The United Nations Children's Fund (UNICEF), the World Health Organization (WHO), and other international health organizations to prevent a potential outbreak that could jeopardize the global polio eradication program.

## Discussion

Gaza's overcrowded living conditions, combined with inadequate sanitation and healthcare facilities, create an ideal environment conducive to the rapid spread of the virus. Such conditions have historically fueled polio outbreaks linked to contaminated water sources. In 2021, Nigeria similarly faced a resurgence of polio due to circulating vaccine-derived poliovirus (cVDPV) in areas with poor sanitation (2). This reemergence was attributed to low vaccination coverage and inadequate water, sanitation, and hygiene (WASH) infrastructure, which allowed the virus to circulate and mutate (3).

In Gaza, the situation is more severe. In 2022, Gaza had high vaccination coverage-at ~99%—with polio nearly being eradicated (1). However, the escalation of hostilities has led healthcare services to collapse, making comprehensive vaccination campaigns difficult to undertake. Close to 17,000 children have missed routine vaccinations, leaving them vulnerable (4). The few operating health facilities are only partially functional, and there is a dire shortage of medical supplies and vaccines. Humanitarian efforts, such as those by UNICEF and the WHO, are essential but are hampered by the security situation and access problems.

Wars and conflicts create fertile ground for infectious diseases to spread, through the destruction of health infrastructure, contamination of water sources, and the displacement of people. This opens up avenues for outbreaks of many largely preventable diseases, as historically observed in other nations in conflict-Iraq, Syria, and Yemen-with reemergence of polio, cholera, and measles in the wake of vaccination disruption and poor sanitary conditions (5).

Preventing a devastating polio outbreak in Gaza requires immediate, coordinated efforts from global health organizations, governments, and non-profits. The top priority must be improving WASH infrastructure, as inadequate access to clean water and poor sanitation significantly increase the risk of virus transmission. In Gaza, over 1.6 million civilians lack access to clean water, making it difficult to maintain basic hygiene and dramatically increasing the risk of waterborne diseases (6). Furthermore, intensified surveillance and vaccination efforts should target all children under 5 years of age and will require support from the international community for vaccine supply and logistical assistance. Cross-border entry of medical supplies and vaccines into Gaza must be made possible against the background of ongoing conflict. Strengthening Gaza's health infrastructure is also important to contain the virus—this includes equipping and training health personnel to effectively screen and isolate polio cases, as well as conducting public awareness campaigns on the importance of vaccination and hygiene practices to prevent polio transmission.

## Conclusion

The presence of the polio virus in Gaza's sewage is a grave indicator of the region's vulnerability and the urgent need for comprehensive public health interventions. Without swift and decisive action, and the provision of essential aid, a polio outbreak could erupt, leaving the health and lives of Gaza's population hanging in the balance.
